# A Novel Hybrid Model for Automatic Non-Small Cell Lung Cancer Classification Using Histopathological Images

**DOI:** 10.3390/diagnostics14222497

**Published:** 2024-11-08

**Authors:** Oguzhan Katar, Ozal Yildirim, Ru-San Tan, U Rajendra Acharya

**Affiliations:** 1 Department of Software Engineering, Firat University, Elazig 23119, Turkey; okatar@firat.edu.tr; 2 National Heart Centre Singapore, Singapore 169609, Singapore; tanrsnhcl@gmail.com; 3 Duke-NUS Medical School, Singapore 169609, Singapore; 4 School of Mathematics, Physics, and Computing, University of Southern Queensland, Springfield, Ipswich, QLD 4300, Australia; rajendra.acharya@unisq.edu.au; 5 Centre for Health Research, University of Southern Queensland, Springfield, Ipswich, QLD 4300, Australia

**Keywords:** histopathological images, vision transformer, lung cancer, feature extraction, automated diagnosis

## Abstract

**Background/Objectives**: Despite recent advances in research, cancer remains a significant public health concern and a leading cause of death. Among all cancer types, lung cancer is the most common cause of cancer-related deaths, with most cases linked to non-small cell lung cancer (NSCLC). Accurate classification of NSCLC subtypes is essential for developing treatment strategies. Medical professionals regard tissue biopsy as the gold standard for the identification of lung cancer subtypes. However, since biopsy images have very high resolutions, manual examination is time-consuming and depends on the pathologist’s expertise. **Methods**: In this study, we propose a hybrid model to assist pathologists in the classification of NSCLC subtypes from histopathological images. This model processes deep, textural and contextual features obtained by using EfficientNet-B0, local binary pattern (LBP) and vision transformer (ViT) encoder as feature extractors, respectively. In the proposed method, each feature matrix is flattened separately and then combined to form a comprehensive feature vector. The feature vector is given as input to machine learning classifiers to identify the NSCLC subtype. **Results**: We set up 13 different training scenarios to test 4 different classifiers: support vector machine (SVM), logistic regression (LR), light gradient boosting machine (LightGBM) and extreme gradient boosting (XGBoost). Among these scenarios, we obtained the highest classification accuracy (99.87%) with the combination of EfficientNet-B0 + LBP + ViT Encoder + SVM. The proposed hybrid model significantly enhanced the classification accuracy of NSCLC subtypes. **Conclusions**: The integration of deep, textural, and contextual features assisted the model in capturing subtle information from the images, thereby reducing the risk of misdiagnosis and facilitating more effective treatment planning.

## 1. Introduction

Cancer is characterized by aberrant unregulated proliferation of cells within the body [1]. Normally, cells undergo a specific and ordered sequence of growth, division, and death [2]; in cancer, this process is disrupted, resulting in uncontrolled and rapid growth of cells [3]. Despite recent advances in cancer research, cancer remains a leading cause of death and a public health concern [4]. According to 2022 GLOBOCAN data [5], lung cancer is the most commonly diagnosed cancer and the leading cause of cancer deaths globally, accounting for 12.4% of all cancers, approximately 2.5 million incident cases annually, and approximately 1.8 million deaths (18.7% of all cancer deaths).

Lung cancer is histologically classified into small cell lung cancer (SCLC) and non-small cell lung cancer (NSCLC) [6], with the latter, which include adenocarcinoma and squamous cell carcinoma [7], accounting for 80–85% of cases [8]. Within this, 25–30% of patients with stage IV NSCLC die within three months of diagnosis [9]. Treatment options for lung cancer range from chemotherapy to targeted therapies [10,11]: the type of treatment is determined by the cancer cell type on tissue biopsy [12]. Tissue samples taken during the biopsy are preserved in a solution, coated with paraffin and cut into thin slices [13]. Tissue sections are typically stained with hematoxylin and eosin (H&E) [14], and the images are digitized for expert analysis [15]. Manual pathological reading is time-consuming and operator-dependent [16]. Furthermore, human factors such as fatigue and distraction may introduce errors, which can negatively affect treatment decisions [17].

To mitigate erroneous diagnosis, computer-aided diagnostic methods have been developed to help pathologists screen and examine histopathological images [18,19,20], including lung cancer [21,22]. As the images are typically acquired with high-resolution scanners at 20× or 40× magnifications [23], file sizes are often several gigabytes [24], which can increase the computational demands of image feature extraction. Nevertheless, computer-aided methods for image analysis are particularly effective for handling datasets that are challenging to analyze manually [25], can be very efficient and accurate [26], and can help reduce the workload of pathologists and speed up the diagnostic process. By improving accuracy and minimizing misdiagnoses, they play a significant role in transforming pathology workflow, ensuring faster and more precise diagnoses [27]. Moreover, computer-assisted systems help decrease variations, standardize procedures, and enhance overall healthcare quality by improving treatment planning [28].

Researchers often focus on isolated aspects of feature extraction, such as deep or textural features. For example, Ebrahim and Fathi used the EfficientNet-B0 model to classify lung cancer on histopathological images [29]. Their study dataset comprised 200 H&E-stained histopathological images of lung cancer tissues scanned at 20× magnification, which had been sourced from four pathology laboratories. Each whole slide image (WSI) was divided into patches of 256 × 256 pixels. Patches were labeled as “tumor” if more than 75% of the pixel area showed cancer features; otherwise, they were labeled “normal”. In total, 12,000 patches were analyzed, which included an equal number of normal and tumor patches. To ensure data stability, k-fold cross-validation was employed, with the model trained over five folds and 100 epochs. The model attained an average accuracy of 92.58%. Ahmed et al. proposed a deep learning model for analyzing histopathological slides for lung cancer [30] that encompassed convolutional neural networks (CNNs) and separable CNNs with residual blocks to enhance classification performance. The dataset included slides from adenocarcinoma patients with mixed (micropapillary, solid and acinar) subtypes. A total of 170 WSIs stained with H&E were studied, from which an average of six images per slide were extracted through zooming and rotation, resulting in 934 images. These images were categorized into 557 cancer and 377 non-cancer images. They were divided into training and test datasets using a 70/30 split ratio. The training dataset included 401 cancer and 261 non-cancer images, with 90% used for model training and 10% for validation. The test dataset comprised 156 cancer images and 116 non-cancer images. The method attained 97.00% accuracy on the test samples. An et al. proposed an approach for lung cancer classification from WSIs using a transformer-based weakly supervised learning framework [31]. Their dataset comprised 646 WSIs from 257 patients diagnosed with adenocarcinoma and pulmonary sclerosing pneumocytoma. These tissues were stained with H&E and scanned at 40× magnification. The WSI images, with an average size of 125,259 × 97,272 pixels at the highest magnification, were stored in a multi-resolution pyramid format consisting of eight different resolution levels. The model attained 96.90% accuracy.

We observed that there is a lack of models that integrate multiple types of features. Single-feature models often struggle with data variations and may not generalize well across different datasets. This can result in missing important details and incomplete image analysis, leading to suboptimal classification performance and a higher risk of misdiagnosis, hence limiting their practical application. To address this gap, we proposed in this study a novel hybrid model that can automatically classify different types of NSCLC from histopathological images. The proposed model combines deep features extracted using the EfficientNet-B0, textural features extracted from grayscale images using the local binary pattern (LBP), and contextual features extracted using the vision transformer (ViT) encoder. By merging these feature sets into a single feature vector, the proposed model achieved higher classification accuracy with classifiers, namely, support vector machine (SVM), logistic regression (LR), light gradient boosting machine (LightGBM), and extreme gradient boosting (XGBoost).

The main contributions of this study can be summarized as follows:

- NSCLC classification was performed with a hybrid model that uses deep, textural and contextual fused features.
- To the best of our knowledge, this study is the first to merge LBP and CNN-based structures with a transformer-based architecture.
- Comprehensive experiments have been conducted to analyze the impact of each feature type on classification accuracy.
- The proposed method can be employed to automatically classify other types of cancer images.

The remainder of this paper is structured as follows: Section 2 describes the materials and methods used, including the dataset and feature extraction techniques. Section 3 presents the experimental setup, performance evaluation metrics and results. Section 4 discusses the results, providing a comparative analysis of different classifiers. Finally, Section 5 offers conclusions and potential directions for future work.

## 2. Materials and Methods

**Figure 1:**
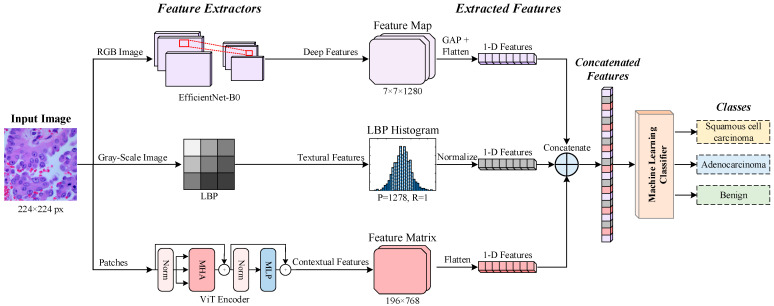
Block diagram of the proposed method for 3-class NSCLC histopathological classification.

In this study, we proposed a hybrid model for automatically classifying lung cancer types from histopathological images (Figure 1). On input images of size 224 × 224 pixels, deep features are extracted from RGB images using EfficientNet-B0, textural features from grayscale images using LBP, and contextual features by feeding input images as patches into the ViT encoder. The extracted deep, textural and contextual feature vectors are merged into a comprehensive combined feature vector, which is fed to a machine learning classifier that performs classification into “lung squamous cell carcinoma (LSCC)”, “lung adenocarcinoma (LACA)”, and “benign” categories.

### 2.1. Feature Extraction Methods

#### 2.1.1. Deep Feature Extraction

**Figure 2:**
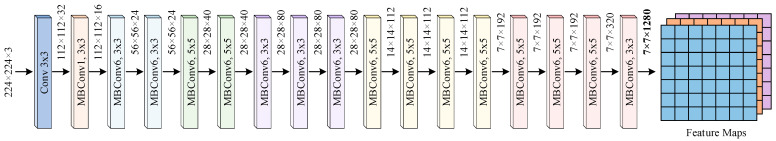
EfficientNet-B0 architecture of mobile inverted bottleneck convolution (MBConv) layers. Here, 224 × 224-pixel RGB histopathological images are input to the network, which processes the images via a series of convolutional layers, each applying various filters to the input data that can identify and learn different spatial hierarchies and complex patterns in the images. The model’s lower layers detect basic features like edges and textures, while the higher layers build on these initial features, allowing the model to recognize more complex and abstract patterns. The final convolutional layer produces feature maps, which are high-level representations of the input images that capture critical patterns and structures required for further processing or classification tasks.

The EfficientNet family encompasses a series of models designed to enhance scalability in CNNs. These models utilize a novel approach to scaling, compound scaling, that simultaneously optimizes the size, depth, and resolution of the network [32], thereby maintaining the balance among these three factors [33]. EfficientNet offers models from B0 to B7: B1 to B7 are scaled versions of the base model EfficientNet-B0 [34], which is made up of a series of multiple mobile inverted bottleneck convolution (MBConv) layers [35]. The hierarchical architecture of MBConv layers allows the model to learn and represent input images in an increasingly detailed manner: each convolutional layer extracts and refines the feature maps, which are transformed representations of the original images (Figure 2).

#### 2.1.2. Textural Feature Extraction

**Figure 3:**
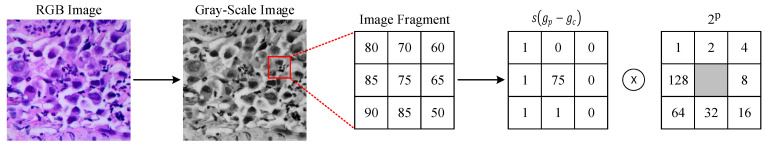
LBP-based feature extraction.

LBP is an effective and computationally efficient feature extraction method used in image processing and pattern recognition [36,37]. LBP encodes the texture of an image by representing each pixel with a binary pattern derived from the intensity values of its neighboring pixels [38] (Figure 3). The technique is simple and robust, yet is able to capture rich descriptions of the patterns [39].

This method uses a window consisting of a central pixel and its surrounding neighbors is used [40]. The value of the central pixel is compared with the values of the neighboring pixels. If a neighboring pixel’s value is greater than or equal to the central pixel’s value, it is assigned a value of 1; otherwise, it is assigned a value of 0 [41]. This way, the binary pattern formed by the neighboring pixels is converted into a decimal number to obtain the LBP value. The steps for computing LBP are summarized below.

1. Take a grayscale image as input.
2. Select a radius (R) and a specific number of neighboring pixels (P) for each pixel.
3. Compare pixel values with neighboring pixel values.
4. The resulting binary values (0 or 1) create a sequence.
5. Convert this sequence to decimal values to obtain the LBP value.

The mathematical operation for computing the LBP value of a pixel is given in Equation (1).

(1) $${LBP}_{P,\;R}=\sum_{p=0}^{P-1}s\left(g_{p}-g_{c}\right)\;\cdot \;2^{p}$$

where $g_{c}$ represents the value of the pixel; $g_{p}$, gray values of the surrounding pixels; *P*, number of neighboring pixels; *R*, radius of the neighboring pixels; and $s\left(x\right)$, a threshold function defined as follows:

(2) $$s\left(x\right)=\left\{\begin{array}{c} 0\;if\;\;x<0\\ 1\;\;if\;\;x\geq 0 \end{array}\right.$$

#### 2.1.3. Contextual Feature Extraction

**Figure 4:**
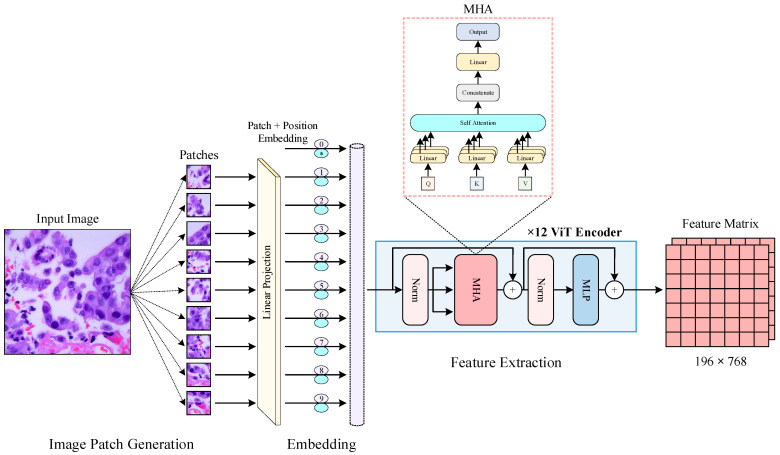
ViT encoder feature extraction architecture used in the study.

Transformer architectures, initially designed for natural language processing tasks, have recently been adapted for image processing applications [42,43]. Dosovitskiy et al. proposed the ViT model, which was specifically adapted for image-processing tasks based on the success of the original transformer architecture [44]. We leveraged the ViT encoder to extract contextual features from histopathological images, which complement the deep and textural features generated by EfficientNet-B0 and LBP, respectively. The ViT encoder architecture consists of multi-head self-attention (MHA) mechanisms and a multilayer perceptron (MLP) head [45] (Figure 4). Each layer processes the input data by computing attention scores that indicate the importance of different parts of the input relative to each other [46]. This mechanism allows the model to understand contextual relationships between different regions of the image, thereby enhancing feature extraction beyond local patterns.

When using the ViT encoder to extract features, the input histopathological images are divided into smaller patches (e.g., 16 × 16 pixels). Each patch is then treated as a “token”, akin to words in a sentence for text processing [47]. Each image patch is flattened and linearly transformed into a fixed-size embedding vector. These embedding vectors are then combined with positional encodings that provide information about the position of each patch within the original image, ensuring that spatial relationships are preserved. The embedded patches are fed into the transformer encoder, where the multi-head self-attention mechanism computes attention scores. Attention scores are calculated using the query (*Q*), key (*K*), and value (*V*) matrices, which are derived from the input embedding vectors. The attention mechanism is formulated as given in Equation (3).

(3) $$Attention\;\left(Q,\;K,\;V\right)=softmax\left(\frac{QK^{T}}{\sqrt{d_{k}}}\right)V$$

These scores determine the relevance of each patch to all other patches, which allows the model to focus on important regions and their contextual interactions. The final output from the ViT encoder comprises refined contextual features for each patch. These features are then concatenated to form a comprehensive contextual feature vector. The multi-head self-attention mechanism computes attention in parallel across multiple heads as given in Equation (4).

(4) $$MultiHead\;\left(Q,\;K,\;V\right)=Concat\left({head}_{1},{head}_{2},\ldots .,{head}_{h}\right)W_{o}$$

Each head is computed as given in Equation (5).

(5) $${head}_{i}=Attention\left({QW}_{Q_{i}},{KW}_{K_{i}},{VW}_{V_{i}}\right)$$

where $W_{Q_{i}}$, $W_{K_{i}}$, $W_{V_{i}}$ are the learnable weight matrices for each head; and $W_{o}$, the output weight matrix.

### 2.2. LC25000 Dataset

**Figure 5:**
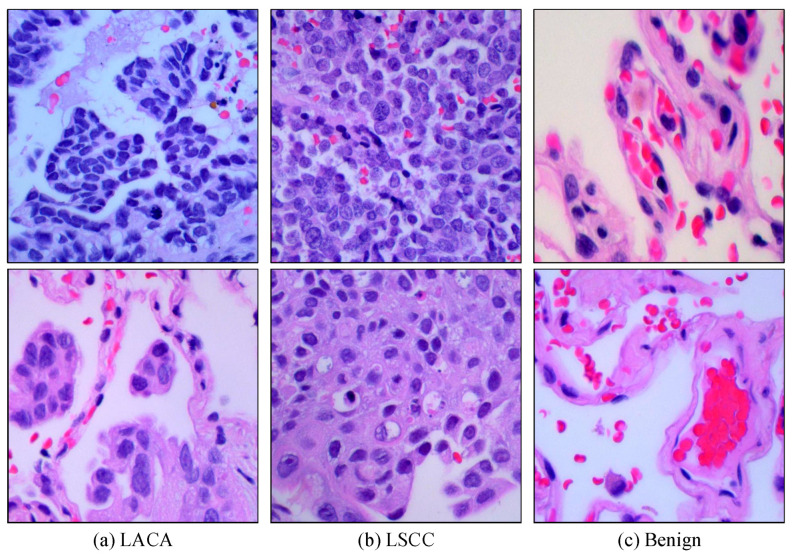
Sample images obtained from the LC25000 dataset: lung adenocarcinoma (LACA) (**a**), lung squamous cell carcinoma (LSCC) (**b**), and benign lung tissue (**c**).

The LC25000 dataset is a collection of histopathological images for lung and colon cancer diagnosis [48]. Each image was originally recorded at a resolution of 1024 × 768 pixels. It was then cropped to 768 × 768 pixels to standardize the dataset. This dataset is divided into five different classes (colon adenocarcinoma, benign colon tissue, lung adenocarcinoma (LACA), lung squamous cell carcinoma (LSCC) and benign lung tissue) of 5000 images each. In this study, we focused on the lung cancer subset of the LC25000. This subset comprises three classes: LACA, LSCC, and benign lung tissue (Figure 5).

### 2.3. Machine Learning Classifiers

Machine learning classifiers are algorithms that classify learning patterns and relationships from datasets [49]. These classifiers are trained using data features extracted using various feature extraction techniques, and they accurately classify new data samples [50]. In this study, we used SVM, LR, LGBM, and XGBoost algorithms to classify features extracted from histopathological images. These algorithms can be summarized as follows.

- SVM is a robust machine learning algorithm recognized for its precision in handling datasets [51]. It maps data points into a space [52]. Identifies the best hyperplane that maximizes the margin between different classes [53].
- LR is a statistical model widely used in classification problems and is particularly effective when the dependent variable is categorical [54]. It predicts the probability of class membership using a linear combination of independent variables.
- LightGBM is an efficient machine learning algorithm effective on large datasets and high-dimensional feature vectors [55]. Based on the gradient boosting framework, it exhibits fast training time and low memory usage [56].
- XGBoost is a widely used, powerful, and flexible gradient-boosting algorithm known for its high accuracy and efficient computation capabilities [57]. It produces effective results on large datasets and complex models by building decision trees sequentially and minimizing errors at each step [58].

## 3. Experimental Results

### 3.1. Experimental Setup

The experiments were conducted using the lung cancer subset of the LC25000 dataset, which contains 15,000 samples. The dataset was randomly split, with 80% of the samples (12,000) used for training and 20% (3000) used for testing. Each image in the dataset was resized from 768 × 768 pixels to 224 × 224 pixels to standardize the input size of the feature extraction methods. Additionally, the images were converted to grayscale to facilitate the extraction of textural features using the LBP (P = 1278, R = 1) method.

**Figure 6:**
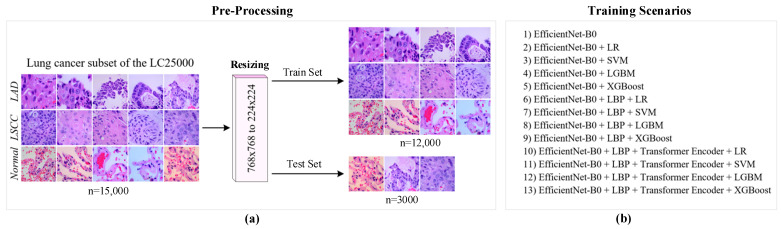
Overview of the experimental setup (**a**) and training scenario (**b**).

The feature extraction process employed a hybrid approach combining deep, textural, and contextual features to comprehensively represent each image. In addition, 13 training scenarios were created using various machine learning classifiers. Each training scenario was tested using performance metrics. All experiments were performed using an NVIDIA RTX 4090 GPU. Figure 6 shows a general overview of the experimental setup.

### 3.2. Performance Evaluation Metrics

The confusion matrix is a tool used to assess the performance of the classification models [59]. It shows the correct and incorrect predictions for each class by the model. The confusion matrix has four components:

1. True Positive (TP): The number of instances correctly predicted by the model as belonging to the class.
2. True Negative (TN): The number of instances correctly predicted by the model as not belonging to the class.
3. False Positive (FP): The number of instances incorrectly predicted by the model as belonging to the class.
4. False Negative (FN): The number of instances incorrectly predicted by the model as not belonging to the class.

**Figure 7:**
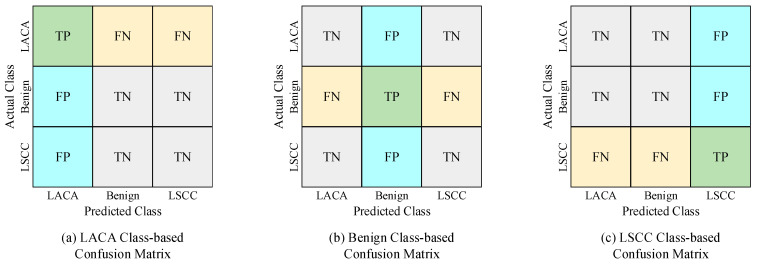
Confusion matrices used in the study: (**a**) LACA Class-based, (**b**) Benign Class-based, and (**c**) LSCC Class-based.

For a multi-class classification model, these metrics are calculated separately for each class [60]. Figure 7 shows the distributions of TP, TN, FP, and FN for the three different classes used in this study.

Various performance metrics are calculated using the components of the confusion matrix. These metrics can be summarized as follows.

Accuracy is defined as the ratio of all correct classifications to the total number of samples. The accuracy rate is calculated using the mathematical equation given in Equation (6).

(6) $$Accuracy=\frac{TP+TN}{\left(TP+FP+FN+TN\right)}$$

Precision indicates the proportion of positive predictions that are actually positive. The precision rate is calculated using the mathematical equation given in Equation (7).

(7) $$Precision=\frac{TP}{TP+FP}$$

Recall represents the proportion of actual positive instances correctly identified by the model. The recall rate is calculated using the mathematical equation given in Equation (8).

(8) $$Recall=\frac{TP}{TP+FN}$$

The F1 Score is the harmonic mean of precision and recall. The F1 Score is calculated using the mathematical expression given in Equation (9).

(9) $$F1\;Score=2\times \frac{Precision\times Recall}{Precision+Recall}$$

These metrics offer an evaluation of the model’s overall effectiveness in recognizing true positives accurately and its capability to minimize false positive outcomes.

### 3.3. Results

**Figure 8:**
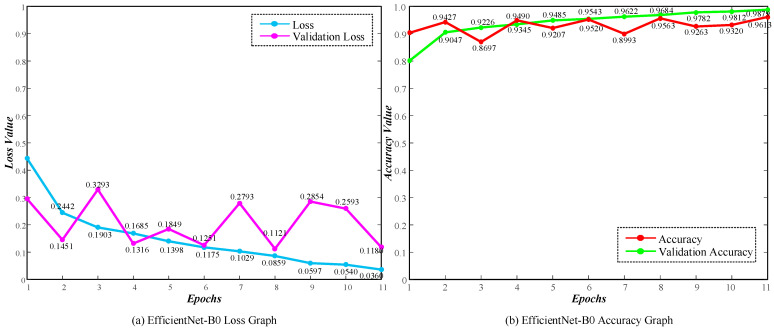
EfficientNet-B0 model performance curves: (**a**) Loss Graph and (**b**) Accuracy Graph.

First, we evaluated the performance of the EfficientNet-B0 model in classifying images of LACA, benign, and LSCC. We configured the model’s final layer to predict outcomes for these three classes. Throughout the training phase, we maintained a learning rate of 0.0001 for 100 epochs. Also, we implemented the early stopping function. This function halted the training process if no improvement in test accuracy was observed over the five epochs. Figure 8 shows the loss and accuracy curves obtained for various epochs of the EfficientNet-B0 model.

The training loss of the EfficientNet-B0 model decreased rapidly from 0.4434 in the first epoch to 0.0360 in the 11th epoch, indicating that the model quickly reduced errors during training. Similarly, training accuracy increased from 80.07% to 98.79%, demonstrating the model’s improved ability to classify training data correctly. Although validation loss decreased from 0.2951 in the first epoch to 0.1180 in the 11th epoch, it exhibited fluctuations in some epochs (e.g., 3rd and 7th epochs). This suggests that the model encountered some challenges during validation. However, validation accuracy increased from 90.30% to 96.13%.

After training with the default layers of the EfficientNet-B0 model, we obtained a test accuracy rate of 96.13%. To explore additional training scenarios involving classical machine learning classifiers, we removed the classifier layer from the EfficientNet-B0 model and treated it as a feature extractor. The output of this feature extractor is a feature map with dimensions of 7 × 7 × 1280.

**Figure 9:**
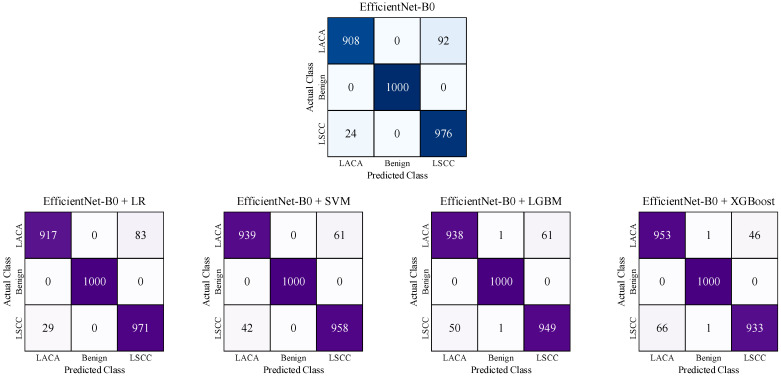
Summary of confusion matrices obtained for various test scenarios using only deep features.

However, classical machine learning classifiers typically require one-dimensional vectors. To convert this 3D feature map into a 1D vector, we passed the model’s output through a Global Average Pooling2D layer. This process averaged each channel, reducing the feature map from 7 × 7 × 1280 to 1 × 1 × 1280. Then, we applied a flattening process to convert the features to a 1D vector. We performed this process separately for 12,000 training images and 3000 test images. Subsequently, we combined the 1280-dimensional vectors obtained from the training samples into a 12,000 × 1280 matrix. Similarly, we repeated the same process for the test samples and obtained a 3000 × 1280 matrix. Using these matrices, we trained and tested the classifiers. The confusion matrices obtained from the test scenarios considering only deep features are given in Figure 9.

The highest test accuracy rate of 96.57% was achieved using the EfficientNet-B0 + SVM hybrid approach. To further enhance the proposed model by incorporating textural features alongside deep features, we extracted an equal number of features using the LBP method. We combined these features with features extracted by EfficientNet-B0.

**Figure 10:**
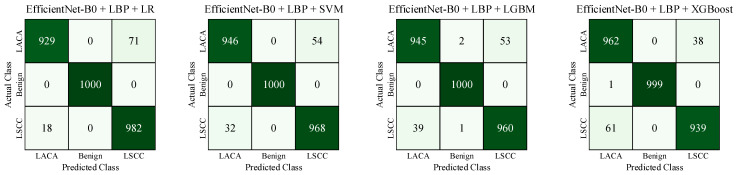
Summary of confusion matrices obtained for various test scenarios using both deep and textural features.

The combined training features from EfficientNet-B0 and LBP were then transformed into a 12,000 × 2560 matrix. Similarly, we repeated the same process for the test samples and obtained a 3000 × 2560 matrix. Using these matrices, we trained and tested the classifiers. The confusion matrices obtained from the test scenarios considering both deep and textural features are given in Figure 10.

The highest test accuracy of 97.13% was achieved using the EfficientNet-B0 + LBP + SVM hybrid approach. We used the ViT encoder to investigate the added value of contextual features in addition to deep and textural features.

**Figure 11:**
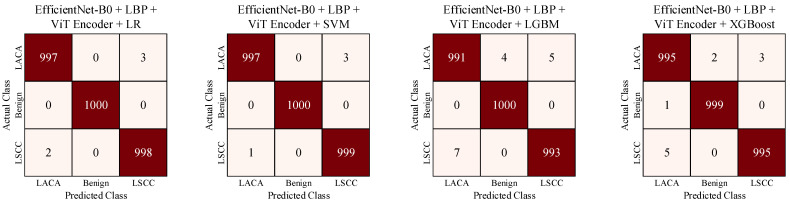
Summary of confusion matrices obtained for various test scenarios using deep, textural, and contextual features.

Using the ViT encoder network, we extracted 196 patches from an input image of size 224 × 224 pixels. Each patch was then converted into a 768-dimensional feature vector by the ViT encoder. By concatenating these feature vectors, we obtained a matrix of size 196 × 768. The matrix was then flattened and combined with deep and textural feature vectors. As a result, we obtained a 12,000 × 153,088 matrix that covers the entirety of the training images. Similarly, we repeated the same process for the test samples, resulting in a 3000 × 153,088 matrix. Using these matrices, we trained and tested the classifiers. The confusion matrices obtained from the test scenarios using deep, textural, and contextual features are given in Figure 11.

In our experiments, the highest test accuracy of 99.87% was achieved using EfficientNet-B0 + LBP + ViT encoder + SVM. The performance values obtained from our experimental studies are summarized in Table 1.

After analyzing the performance of the classifiers, we found that SVM outperforms the others. This superior performance can be attributed to SVM’s robust handling of high-dimensional spaces. Its effectiveness is particularly evident in scenarios characterized by complex feature sets. Moreover, SVM’s intrinsic ability to maximize the margin between classes facilitates a clearer separation of the data, enhancing classification accuracy.

## 4. Discussion

**Figure 12:**
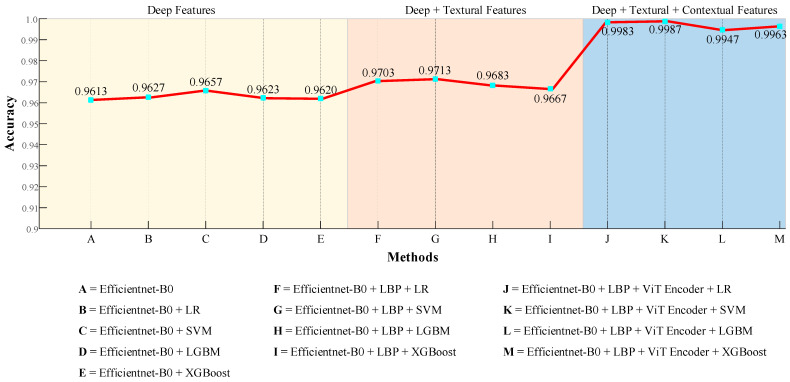
Plot of test accuracies obtained for various combinations.

In this paper, we proposed a hybrid method for the automatic classification of histopathological lung cancer images by leveraging a combination of advanced deep learning and feature extraction techniques. The proposed method integrates EfficientNet-B0 for deep feature extraction, LBP for textural feature extraction, and ViT encoder for contextual feature extraction. The proposed comprehensive method attempts to enhance classification accuracy by capturing various visual features. Figure 12 shows an overview of the test accuracy values obtained for various combinations.

First, we examined the classification accuracy obtained using the EfficientNet-B0 model for LACA, benign and LSCC classes. Although the overall performance of EfficientNet-B0 was satisfactory, we observed an improvement when the model was combined with classifiers. This shows that deep features yield better results when supported by more effective classification methods. When considering combinations of EfficientNet-B0 and LBP, we found that the performance improved even further. The effectiveness of LBP in extracting textural features significantly contributed to improving the classification performance. The proposed hybrid method, which integrates the EfficientNet-B0, LBP, and ViT encoder, yielded the highest performance. Specifically, nearly perfect results were obtained when combined with SVM and LR. This demonstrates the effectiveness of the ViT encoder for feature extraction and classification performance. Hence, ViT encoder helps to learn the complex relationships in the data, thereby boosting the classification accuracy.

Automatic detection of lung cancer using histopathological images is a hot research area. We performed a non-systematic search of the literature for studies conducted using the LC25000 dataset, and summarized our findings in Table 2. Hatuwal and Thapa proposed a CNN model for lung cancer detection using histopathological images [61]. Their model, consisting of an input layer, three hidden layers, and a fully connected layer, achieved an accuracy of 97.20% on test images. Mangal et al. also developed a CNN model for lung cancer detection from histopathological images [62]. Their model consisted of three convolutional layers, three pooling layers, and two fully connected layers. It achieved an accuracy of 97.89% on test images. Baranwal et al. evaluated the performance of several models for classifying lung cancer in histopathological images [63]. The models included ResNet-50, VGG-19, Inception-ResNet-V2, and DenseNet. Among these models, the Inception-ResNet-V2 model demonstrated the highest performance with an accuracy of 99.70%. Javier et al. investigated the impact of using color versus grayscale images on lung cancer detection accuracy [64]. They developed a CNN model with three layers and a max-pooling layer. Their model trained with color images achieved an accuracy of 97.11% on the test samples, which outperformed the other models. Wadekar and Singh introduced a modified CNN model incorporating a pretrained VGG-19 model for classifying lung cancer histopathology images [65]. By implementing data augmentation methods and fine-tuning, their VGG-19 model achieved an accuracy of 97.73% on test samples. Hamed et al. proposed an approach combining a custom CNN model with a LightGBM classifier to categorize lung tissue histopathology images, achieving an impressive accuracy rate of 99.60% on test samples [66]. Mercaldo et al. proposed a deep learning-based technique to automatically detect cells in lung tissue images using CNN, AlexNet, VGG-16, VGG-19, and MobileNet models. Among these, VGG-16 demonstrated the best performance, with an accuracy rate of 99.20% on the test samples [67]. Tian et al. introduced an approach for accurately classifying lung cancer cells using neural networks [68]. Their model merges a feature pyramidal network (FPN) and squeeze-and-excitation (SE) components with a ResNet-18 model to improve image processing capabilities and perform scale analysis for lung cancer categorization. The proposed model achieved a testing accuracy of 98.84%. Noaman et al. explored the efficacy of combining DenseNet201 with color histogram techniques to improve lung cancer classification from histopathological images [69]. Their study applied various machine learning algorithms, including KNN, LightGBM, CatBoost, XGBoost, Decision Trees (DT), Random Forest (RF), Multinomial Naive Bayes (MultinomialNB), and SVM. Their proposed method achieved an accuracy rate of 99.68%.

In this study, we proposed a hybrid model for the automatic classification of lung cancer types using the LC25000 dataset. The proposed method integrates the EfficientNet-B0, LBP, and ViT encoders for feature extraction, and classification using SVM. This hybrid model was developed using 3000 images and achieved an outstanding accuracy of 99.87%.

The advantages of the proposed hybrid model are summarized as follows:

- The proposed model achieved an accuracy of 99.87% surpassing previous studies.
- By combining the EfficientNet-B0, LBP, and ViT encoder, the proposed model effectively captures subtle features. The proposed model can identify both high-level abstract patterns and fine-grained local textures in histopathological images.
- The high classification accuracy of the proposed model provides the potential to be used in real-world clinical applications.
- The modular structure of our model allows for customization to meet various clinical needs and data types. This flexibility makes the proposed method applicable to diagnosing different types of cancer and other medical imaging problems.

The limitation of this work is that the proposed method was evaluated on a single dataset, the LC25000, which consists of pre-processed and standardized histopathological images. However, real-world applications may face inconsistencies in specimen preparation and slide positioning, which could affect model performance. To address this, future work will focus on strategies to incorporate automated quality control measures and adapt the model to handle such variations effectively. The dataset will also be expanded to include rarer cases such as SCLC, and traditional immunohistochemical stains may be considered to improve accuracy, especially for smaller sample sizes.

Future work will explore multi-modal learning techniques that combine histopathological and molecular data to improve classification accuracy in complex cases such as undifferentiated lung cancer. We also plan to investigate the integration of traditional biomarkers such as TTF-1 and P40, aiming to combine morphological and molecular data for enhanced diagnostic performance. Furthermore, efforts will focus on developing user interfaces and software tools to facilitate clinical integration. The explainability of the model’s decision-making process will be improved to ensure better understanding and trust among clinical experts [70]. Pre-processing techniques, such as quality assessment filters and noise reduction, will also be incorporated to mitigate the risk of errors in cases with suboptimal specimen quality.

## 5. Conclusions

In this study, we proposed a hybrid model that integrates the EfficientNet-B0, LBP, and ViT encoder to automatically classify NSCLC subtypes using histopathological images. Our comprehensive feature extraction approach demonstrated superior performance using the SVM classifier. The experimental results indicate that including textural and contextual features significantly enhance the classification accuracy more than deep features alone. The highest test accuracy of 99.87% indicates the potential of the proposed method in accurate diagnosis, thereby reducing the risk of misdiagnosis and facilitating more effective treatment planning for NSCLC patients. This study contributes to the accurate automated lung cancer diagnosis, using multiple feature extraction techniques. Future work may focus on expanding this approach to other cancer types and optimizing the model to handle larger and more diverse datasets. The integration of such advanced diagnostic tools in clinical practice helps in accurate cancer diagnosis.

## Figures and Tables

**Table 1:** Summary of performance values obtained from the experimental studies.

Method	Class	Precision	Recall	F-1 Score	Accuracy	Inference Time (s)
EfficientNet-B0	LACA	0.9742	0.9080	0.9399	0.9613	0.0343
	Benign	1	1	1		
	LSCC	0.9138	0.9760	0.9439		
EfficientNet-B0 + LR	LACA	0.9693	0.9170	0.9424	0.9627	0.0879
	Benign	1	1	1		
	LSCC	0.9212	0.9710	0.9454		
EfficientNet-B0 + SVM	LACA	0.9571	0.9390	0.9480	0.9657	0.0868
	Benign	1	1	1		
	LSCC	0.9401	0.9580	0.9489		
EfficientNet-B0 + LGBM	LACA	0.9493	0.9380	0.9436	0.9623	0.0937
	Benign	0.9980	1	0.9990		
	LSCC	0.9396	0.9490	0.9442		
EfficientNet-B0 + XGBoost	LACA	0.9352	0.9530	0.9440	0.9620	0.0996
	Benign	0.9980	1	0.9990		
	LSCC	0.9530	0.9330	0.9429		
EfficientNet-B0 + LBP + LR	LACA	0.9809	0.9290	0.9542	0.9703	1.1821
	Benign	1	1	1		
	LSCC	0.9325	0.9820	0.9566		
EfficientNet-B0 + LBP + SVM	LACA	0.9672	0.9460	0.9565	0.9713	1.1810
	Benign	1	1	1		
	LSCC	0.9471	0.9680	0.9574		
EfficientNet-B0 + LBP + LGBM	LACA	0.9603	0.9450	0.9526	0.9683	1.1879
	Benign	0.9970	1	0.9985		
	LSCC	0.9476	0.9600	0.9538		
EfficientNet-B0 + LBP + XGBoost	LACA	0.9394	0.9620	0.9505	0.9667	1.1938
	Benign	1	0.9990	0.9995		
	LSCC	0.9611	0.9390	0.9499		
EfficientNet-B0 + LBP + ViT Encoder + LR	LACA	0.9979	0.9970	0.9974	0.9983	1.3110
	Benign	1	1	1		
	LSCC	0.9970	0.9980	0.9975		
EfficientNet-B0 + LBP + ViT Encoder + SVM	LACA	0.9989	0.9970	0.9979	0.9987	1.3099
	Benign	1	1	1		
	LSCC	0.9970	0.9990	0.9980		
EfficientNet-B0 + LBP + ViT Encoder + LGBM	LACA	0.9929	0.9910	0.9919	0.9947	1.3168
	Benign	0.9960	1	0.9980		
	LSCC	0.9949	0.9930	0.9939		
EfficientNet-B0 + LBP + ViT Encoder + XGBoost	LACA	0.9940	0.9950	0.9945	0.9963	1.3227
	Benign	0.9980	0.9990	0.9985		
	LSCC	0.9969	0.9950	0.9959		

**Table 2:** Summary of studies conducted on the classification of NSCLC subtypes using the LC25000 dataset.

Study	Year	Dataset	Number of Classes	Method	CV Ratio	Performance
Hatuwal and Thapa [61]	2020	LC25000	3 (LACA, LSCC and Benign)	Custom CNN	90% Train, 10% Validation	Accuracy = 97.20%
Mangal et al. [62]	2020	LC25000	3 (LACA, LSCC and Benign)	Custom CNN	90% Train, 10% Validation	Accuracy = 97.89%
Baranwal et al. [63]	2022	LC25000	3 (LACA, LSCC and Benign)	Inception-ResNetv2	80% Train, 20% Validation	Accuracy = 99.70%
Javier et al. [64]	2022	LC25000	3 (LACA, LSCC and Benign)	Custom CNN	80% Train, 20% Test	Accuracy = 97.11%
Wadekar and Singh [65]	2023	LC25000	3 (LACA, LSCC and Benign)	VGG-19	80% Train, 20% Test	Accuracy = 97.73%
Hamed et al. [66]	2023	LC25000	2 (LSCC and Benign)	Custom CNN + LightGBM	40% Train, 60% Test	Accuracy = 99.60%
Mercaldo et al. [67]	2024	LC25000	3 (LACA, LSCC and Benign)	VGG-16	80% Train, 10% Validation, 10% Test	Accuracy = 99.20%
Tian et al. [68]	2024	LC25000	3 (LACA, LSCC and Benign)	FPN + SE + ResNet-18	N/A	Accuracy = 98.84%
Noaman et al. [69]	2024	LC25000	3 (LACA, LSCC and Benign)	DenseNet-121 + Color Histogram + KNN	N/A	Accuracy = 99.68%
This study	2024	LC25000	3 (LACA, LSCC and Benign)	EfficientNet-B0 + LBP + ViT Encoder + SVM	80% Train, 20% Test	Accuracy = 99.87%

## Data Availability

Data are available in a publicly accessible repository. The original data presented in the study are openly available in Academic Torrents at https://academictorrents.com/details/7a638ed187a6180fd6e464b3666a6ea0499af4af, accessed on 23 June 2024.
